# The Relationship Between Occupational Functionality and Metacognition in Patients with Obsessive-Compulsive Disorder

**DOI:** 10.7759/cureus.51738

**Published:** 2024-01-06

**Authors:** Esra Yalım, Cansu Ünsal, İbrahim Gündoğmuş

**Affiliations:** 1 Psychiatry, Çankırı State Hospital, Çankırı, TUR; 2 Psychiatry, Silifke State Hospital, Mersin, TUR; 3 Psychiatry, Ankara Etlik City Hospital, Ankara, TUR

**Keywords:** negative beliefs, metacognition, positive beliefs, obsessive-compulsive disorder, occupational functionality

## Abstract

Objective: Obsessive-compulsive disorder (OCD) is one of the most common mental disorders with a loss of functionality in many areas of life. The current study aims to reveal the relationship between occupational functionality and metacognition in OCD patients.

Materials and methods: The cross-sectional study sample consisted of 183 participants diagnosed with OCD according to the Diagnostic and Statistical Manual of Mental Disorders, Fifth Edition (DSM-5) criteria. Participants were evaluated with the Yale-Brown Obsession and Compulsion Scale (Y-BOCS), Beck Depression Inventory (BDI), Beck Anxiety Inventory (BAI), and Metacognition Scale (MCQ-30). The occupational functioning of the participants was determined by the basic version of the semi-structured Longitudinal Follow-up Evaluation form (LIFE-BASE) in interviews with the clinician. The LIFE-BASE form divided the study sample into functional (n=92) and non-functional (n=91).

Results: It was determined that the mean age of the study sample was 32.49±8.65 years, and 54.1% (n=99) of them were female. When the two groups were compared, statistically significant differences were found in gender (p<0.001), education (p=0.012), Y-BOCS compulsion (p=0.003), and total scores (p=0.006). In the comparison of the groups, a statistical difference was found between the MCQ-30 Positive Beliefs subscale (p<0.05). However, no statistical difference was found between uncontrollability and danger, cognitive confidence, belief in controlling thoughts, and cognitive awareness. In addition, it was found that gender and the MCQ-30 positive beliefs score could predict occupational functioning.

Conclusion: The present study is important because it was the first study to show the effects of positive beliefs on occupational functioning in OCD patients. However, further studies are needed on the underlying causes of this effect.

## Introduction

Obsessive-compulsive disorder (OCD) is characterized by the presence of obsessions, compulsions, or both, and its lifetime prevalence is between 2% and 3% [1]. It is known that OCD is a chronic mental disorder that negatively affects a person's family, academic, professional, and social functionality and quality of life [2].

Metacognition refers to an individual's capacity to manage and understand their mental processes [3]. Metacognition means being aware of one's thought processes and understanding the patterns behind these processes. Metacognition can take many forms, such as reflecting on one's thought patterns and knowing when and how to use certain strategies for problem-solving [4].

According to the metacognitive approach, this system contributes to the development and maintenance of psychopathology by causing maladaptive reaction forms in the individual. In the metacognitive model of OCD, individuals base their negative comments on intrusive thoughts and their beliefs about the importance and impact of such thoughts. Due to metacognitions based on inappropriate criteria, the person performs some neutralizing and controlling rituals. This leads to the continuity of their evaluation of the threat and a decrease in their confidence in their memory [3]. Lenzo et al. stated that metacognition is associated with anxiety, depression, quality of life, and functionality in individuals with chronic diseases [5].

In the previous literature examined it was found that there were a limited number of studies on the level of occupational functionality in OCD patients. In a study conducted in this context, the participants reported that 52.9% did not have sufficient occupational functionality [6]. An epidemiological study by Ruscio et al. indicated that the disorder caused an average of 46 days of work productivity in the past year in OCD patients [7]. In another study in which functionality was extensively evaluated, it was determined that patients with OCD had more impairment in functionality than patients with major depressive disorder but showed little difference from patients with inpatient schizophrenia [8]. The authors suggested that variables such as gender, age at onset, duration of OCD, symptom subtypes, and accompanying psychiatric conditions may be related to occupational functionality [9]. It is necessary to investigate the processes that affect functionality in OCD patients, where functionality is noted as an important problem.

Although there are various studies on metacognitive features in OCD patients in the literature, no study has been found investigating the relationship between metacognition and occupational functionality. The aim of the current study is to investigate the correlation between metacognition and occupational functionality levels in patients with OCD.

## Materials and methods

Participants

The cross-sectional study included 183 patients diagnosed with OCD based on the Diagnostic and Statistical Manual of Mental Disorders, Fifth Edition (DSM-5) criteria who sought treatment at Gulhane Training and Research Hospital, Ankara, Turkey, and Kırıkkale Yüksek İhtisas Hospital Psychiatry outpatient clinic, Kırıkkale, Turkey, between February and November 2021. Twenty-two patients who did not meet the inclusion criteria or did not sign the informed consent form for participation in the study were excluded. Ethical approval was granted by the ethics committee of the University of Health Sciences, Gülhane Training and Research Hospital Clinical Studies, Ankara, Turkey (approval number: 25.02.2021-2021.01.22).

This study included individuals between the ages of 18 and 65 who were diagnosed with OCD according to the DSM-5 criteria. Patients with cognitive impairment, a history of neurological disease, head trauma, mental retardation, bipolar disorder, psychotic disorder, a history of substance abuse, and those who were unable to read or write were excluded. Also, patients without an occupation (homemakers, retired patients, or students) with a physical disease that would prevent employment were not included. In addition, the patients had not been on psychotropic medication for their disease at the time of inclusion in the study.

Instruments for data collection

Sociodemographic Data Collection Form

Developed by the researchers to analyze data, the form considered the characteristics of the patient group in alignment with the study's aims and assumptions. In the form, participants' age, gender, education level, marital status, and economic status were questioned.

Longitudinal Follow-up Evaluation Form (LIFE-BASE)

The occupational functionality of the participants was evaluated with the basic version of the LIFE-BASE form [10]. The LIFE-BASE form, a semi-structured interview, is a seven-point Likert-type scale that evaluates professional functionality in the past month in psychiatric disorders. Lack of professional functionality in work was defined as the inability to work due to OCD-related psychopathology. According to the LIFE-BASE scale, those who received the code 0 (0 = not applicable; did not study for any reason other than psychopathology, e.g., student, homemaker, etc.) and code 6 (6 = no information) were excluded from the study. The LIFE-BASE scale is accepted as "professional functionality" in codes 1, 2, and 3 and "no professional functionality" in codes 4 and 5.

Yale-Brown Obsessive Compulsion Scale (Y-BOCS)

The Y-BOCS, a semi-structured scale administered by the interviewer, was developed by Goodman et al. [11]. Although the scale consists of 19 items, the first 10 items are used to calculate the total score. The total score is evaluated from 0 to 40 on a five-point Likert-type scale ranging from 0 to four. Three different evaluation scores can be calculated on the scale: obsession, compulsion, and total score. The Turkish validity and reliability study of the scale was performed by Karamustafalıoğlu et al. The Cronbach's alpha value for the scale was calculated as 0.98 [12].

Beck Anxiety Inventory (BAI)

The scale used to measure the severity of patients' anxiety-related symptoms was developed by Beck et al. in 1988 [13]. Each item is scored between 0 and three on the scale, consisting of 21 items in total, with the highest possible score of 63. A same-way correlation was found between the total score obtained on the scale and the level of anxiety. The Turkish validity and reliability study of the scale was performed by Ulusoy et al. The Cronbach's alpha value for the scale was calculated as 0.93 [14].

Beck Depression Inventory (BDI)

Each item is scored between 0 and three on the BDI, a 21-item self-report scale developed by Beck et al. in 1988 to determine the depression risk of patients and measure the severity of depressive symptoms [15]. There is a correlation in the same direction between the score obtained and the severity of depression. The Turkish validity and reliability study of the scale was performed by Hisli. The Cronbach's alpha value for the scale was calculated as 0.80 [16].

Metacognition Scale (MCQ-30)

The scale was developed by Cartwright-Hatton and Wells and consists of five factors. These factors are positive beliefs, cognitive confidence, uncontrollability and danger, cognitive awareness, and the need for control. The scale is a four-point Likert type, and the total score varies between 30 and 120 points [3]. The Turkish validity and reliability study of the scale was conducted by Tosun et al. An increase in the total score indicates an increase in pathological metacognitive activity. The Cronbach's alpha value for the scale was calculated as 0.86 [17].

Procedure

Patients who applied to the psychiatry outpatient clinic during the study dates and were diagnosed with OCD according to the DSM-5 diagnostic criteria after the clinician examination were invited to the study consecutively. According to the inclusion and exclusion criteria, the OCD symptom severity of the volunteer participants was determined by Y-BOCS. The interviewer evaluated the participants' occupational functionality with the LIFE-BASE form. Then the BAI and BDI for accompanying anxiety and depression symptoms were filled in by the patient. Finally, MCQ-30 was given to the participants. Then, the patients were divided into two groups according to their occupational functionality scores: those having occupational functionality and those not having occupational functionality. Statistical analysis was performed after the obtained data were duly recorded in the data set.

Statistical analysis

Statistical analyses of the study were conducted with IBM SPSS 22.0 (IBM Corp., Armonk, NY) and the Jamovi package program (The jamovi project (2023). jamovi (Version 2.3) (Computer Software) Retrieved from https://www.jamovi.org). The Kolmogorov-Smirnov test was used to determine the normality of the distribution. Descriptive analyses were presented as frequency and percentage for categorical variables and mean and standard deviation for continuous variables. Pearson's Chi-square analysis was used to compare categorical data between the two groups, and the Student's t-test was used after testing parametric assumptions for comparing continuous variables. Univariate and multivariate logistic regression analyses were used to determine predictors of occupational functioning. In all analyses, p<0.05 was considered statistically significant.

## Results

It was determined that 99 (54.1%) of the participants were female, and 84 (45.9%) were male. Information, including sociodemographic data and clinical variables, is presented in Table 1.

**Table 1:** Comparison of the participants' sociodemographic and psychometric data according to occupational functionality groups a: Student's t-test, b: chi-square test; *p<0.05

	Total (n=183)	No occupational functionality (n=91)	Occupational functionality (n=92)	Statistics	p-value
Age (Median±SD)	32.49±8.65	32.99±10.62	32.00±6.13	0.770 ^a^	0.442
Gender (%)					
Female	99 (54.1)	71 (71.7)	28 (28.3)	41.720	<0.001^*^
Male	84 (45.9)	20 (23.8)	64 (76.2)		
Education (%)					
Primary	34 (18.4)	14 (41.2)	20 (58.8)	8.832	0.012^*^
High school	67 (36.6)	43 (64.2)	24 (35.8)		
University	82 (44.8)	34 (41.5)	48 (58.5)		
Marital status (%)					
Married	100(54.6)	52 (52.0)	48 (48.0)	0.456	0.500
Single	83(45.4)	39 (47.0)	44 (53.0)		
Income (%)					
Low	44 (24.0)	20 (45.5)	24 (54.5)	0.478	0.787
Medium	75 (41.0)	39 (52.0)	36 (48.0)		
High	64 (35.0)	32 (50.0)	32 (50.0)		
Yale-Brown Obsession and Compulsion Scale (Y-BOCS) (Median±SD)					
Obsession	12.90±3.00	13.28±2.73	12.52±3.21	1.730^a^	0.085
Compulsion	12.37±3.79	13.19±3.30	11.56±4.08	2.974^a^	0.003^*^
Total	25.27±5.91	26.48±5.44	24.08±6.14	2.790^a^	0.006^*^
Metacognition Scale (MCQ-30) (Median±SD)					
Positive beliefs	13.84±4.63	12.80±4.69	14.86±4.37	-3.084^a^	0.002^*^
Uncontrollability and danger	17.09±4.19	17.50±4.24	16.69±4.13	1.307^a^	0.193
Cognitive confidence	14.75±6.64	14.94±4.30	14.56±4.96	0.553^a^	0.581
Need to control thoughts	19.12±3.89	19.07±3.59	19.17±4.17	-0.168^a^	0.867
Self-consciousness	18.53±3.61	18.41±3.65	18.65±3.59	-0.438^a^	0.662
Total	83.35±13.04	82.74±11.69	83.95±14.07	-0.626^a^	0.581
Beck Depression Inventory (BDI) (Median±SD)	21.49±12.78	24.01±11.93	19.00±13.16	2.699^a^	0.008^*^
Beck Anxiety Inventory (BAI) (Median±SD)	24.68±16.37	26.04±13.74	23.34±18.60	1.116^a^	0.266

Ninety-one (49.7%) of the 183 patients reported impaired functionality due to their psychopathology in the past month. In this context, they were categorized as having "no occupational functionality." When the data on occupational functionality and gender were examined, 71% (n=71) of the female participants and 23.8% (n=20) of the male participants were considered to have no occupational functionality. There was a statistically significant difference between the two groups in terms of gender (p<0.001). In addition, a statistically significant difference was found between the two groups in terms of educational status (p<0.05). There was no statistically significant difference between the two groups in marital status or income level. A statistical difference was found between the two groups in Y-BOCS compulsion (p=0.003) and total (p=0.006) scores. There was no statistical difference in Y-BOCS obsession scores between the two groups.

A significant difference was found between the two groups in the MCQ-30 positive beliefs sub-dimension. Positive beliefs were found to be higher in the group evaluated as having no occupational functionality (12.80±4.69) compared to the group evaluated as having occupational functionality (14.86±4.37) (p=0.002). There was no significant difference between the two groups in the subscales of uncontrollability and danger, cognitive confidence, need to control thoughts, and cognitive awareness of MCQ-30. The two groups had no significant difference in BDI and BAI total scores.

Occupational functionality predictors for OCD patients were examined using the logistic regression model (Table 2).

**Table 2:** Predictors of occupational functioning: univariable and multivariable logistic regression analysis OR: odds ratio

Variables	Univariate			Multivariate		
	B	p-value	OR (95% Cl)	B	p-value	OR (95% Cl)
Age	-0.013	0.439	0.987 (0.954-1.021)			
Gender (female)	2.094	<0.001	8.114 (4.169-15.792)	1.891	<0.001	6.627 (3.266-13.611)
Marital status (married)	0.201	0.500	1.222 (0.682-2.189)			
Education (primary school)	0.357	0.306	1.429 (0.722-2.828)			
Yale-Brown Obsession and Compulsion Scale (Y-BOCS) total	-0.071	0.007	0.931 (0.885-0.981)	-0.023	0.471	0.977 (0.917-1.041)
Beck Depression Inventory (BDI)	-0.032	0.009	0.969 (0.946-0.992)	-0.005	0.755	0.995 (0.966-1.025)
Beck Anxiety Inventory (BAI)	-0.010	0.266	0.990 (0.972-1.008)			
Metacognition Scale (MCQ-30)						
Positive belief	0.100	0.003	1.106 (1.035-1.182)	0.076	0.040	1.079 (1.004-1.160)
Uncontrollability and danger	-0.047	0.193	0.955 (0.890-1.024)			
Cognitive confidence	-0.018	0.579	0.982 (0.923-1.046)			
Need to control thoughts	0.006	0.866	1.006 (0.934-1.085)			
Self-consciousness	0.018	0.660	1.018 (0.940-1.104)			

In the univariate model, the differences between gender (odds ratio (OR): 8.114, 95% CI: 4.169-15.792, p<0.001), Y-BOCS (OR: 0.931, 95% CI: 0.885-0.981, p=0.007), BDI (OR: 0.969, 95% CI: 0.946-0.992, p=0.009), and positive belief (OR: 1.106, 95% CI: 1.035-1.182, p=0.003) were statistically significant. In the multivariate logistic regression model (X2=48.548, df=4, p<0.001, R2=0.311), the differences between gender (OR: 6.627, 95% CI: 3.266-13.611, p<0.001) and positive belief were statistically significant.

The predictors of occupational functioning in OCD patients were examined using the logistic regression model (Table 2). In the univariate model, gender (OR: 8.114, 95% CI: 4.169-15.792, p<0.001), Y-BOCS (OR: 0.931, 95% CI: 0.885-0.981, p=0.007), BDI (OR: 0.969, 95% CI: 0.946-0.992, p=0.009), and positive belief (OR: 1.106, 95% CI: 1.035-1.182, p=0.003) were statistically significant. In the multivariate logistic regression model (X2=48.548, df=4, p <0.001, R2=0.311), gender (OR: 6.627, 95% CI: 3.266-13.611, p<0.001) and the differences between positive belief (OR: 1.079, 95% CI: 1.004-1.160, p=0.040) were statistically significant.

## Discussion

The main result of the current study examining the relationship between occupational functionality and metacognition in OCD patients is that gender, positive beliefs about metacognition, and compulsion levels, which are components of psychopathology, are different between individuals with and without occupational functionality. In addition, positive beliefs about gender and metacognition in OCD patients were found to predict occupational functioning. These results may be useful in planning treatments for OCD patients, for whom reduced functionality is an important problem.

As a result of the data obtained related to metacognition, it was shown that MCQ-30 total scores were significantly higher in OCD patients [18]. Previous studies found that the sub-dimensions of metacognition, the uncontrollability of anxiety, and the need to control negative beliefs and thoughts about its danger were strongly associated with OCD symptoms [19, 20]. Although there was no significant difference between the two groups in the sub-dimension of uncontrollability and danger, this sub-dimension was higher in the group with no occupational functionality, as expected. The uncontrollability and danger sub-dimension, which has a strong relationship with OCD symptoms, may be related to occupational functioning, which is one of the possible indicators of the symptom severity of the disease.

However, there is a need for prospective studies with other sample groups to examine this relationship. The need to control thoughts, positive beliefs, and cognitive awareness sub-dimensions of MCQ were significant predictors of OCD symptoms [21]. The positive beliefs sub-dimension was significantly higher in the group with occupational functionality. Positive metacognitions include rumination, worry, attentional biases, and beliefs that dysfunctional coping behaviors work. Negative metacognitions, on the other hand, emerge following positive metacognitions when considered over time. They include beliefs that focus on the harmful effects of processes that come into play due to positive metacognitions because they cannot be controlled gradually [22]. In other words, positive beliefs in metacognitive structure give way to negative beliefs over time. For this reason, although high scores on positive beliefs in the group characterized as having occupational functionality seem to be the opposite of what was expected, it can be argued that since negative beliefs have not been activated yet, individuals' high functionality and mental preoccupation with anxiety may lead to an increase in productivity by focusing more on work.

Patients with OCD have high unemployment rates and poor functionality. In the present study, being male and having positive beliefs were predictors of occupational functioning. In the study of Coban et al., being male was also a predictor of occupational functionality [6]. Roles attributed to women in business life, differences in expectations from individuals, and stigma against psychiatric diseases may have led to this result. People with a high level of positive belief may be more likely to be recognized more rigorously and regularly in business life, and it is thought that this situation can be seen as a more positive element in terms of professional functionality. Due to the fact that negative beliefs can replace positive beliefs over time, it is necessary to evaluate the occupational functioning of individuals in order to be able to make more precise comments.

It is known that OCD patients have impaired functionality in various areas of life [23]. In this context, in our study, in which occupational functionality was evaluated, it was determined that approximately half of the patients did not have occupational functionality. Similarly, in a study conducted by Coban et al. in Turkey, this rate was 52.9% [6]. The study by Mancebo et al. determined that 38% of OCD patients had inadequate occupational functionality [9]. In another study conducted in a sample of Turkey, the unemployment rate among OCD patients was found to be 67.5% [24]. In a longitudinal study by Pinto et al. on the clinical course and symptoms of OCD patients, it was found that 41% of the participants were unemployed at the time they were included in the study [25]. In a study by Rodrigues-Salgado et al. in Spain on the factors affecting the perceived quality of life in OCD patients, 64 patients determined that 40.6% were unemployed [26]. When the studies on occupational functionality levels in OCD patients were reviewed in the literature, it was seen that there were proportional differences between the data obtained. The fact that the studies were conducted in different countries and with different measurement tools can be shown as the reason for this difference in rates. The occupational functionality data of the present study are similar to the study of Coban et al. [6]. This similarity can be associated with the determination of occupational functionality with the LIFE-BASE form in both studies, the fact that the centers where the study was conducted are located in the city center, and the characteristics of the sample included.

In the study of Coban et al., being a woman, being single, and having a history of suicide attempts were associated with lower occupational functionality, and 40% of the female participants were found to have insufficient occupational functionality [6]. In the study of Mancebo et al., 52.2% of the female participants were found to have insufficient occupational functionality [9]. In the current study, the rate of women was 71.7% in the group evaluated as having no occupational functionality, a higher rate than in both studies. According to the data of the Turkish Statistical Institute for 2021, the labor force participation rate of individuals over the age of 15 in Turkey is 51.4%; this rate was found to be 32.8% in women and 70.3% in men. According to the same data, it was observed that as the education level of women increased, the labor force participation rate increased [27]. Studies conducted in this context may show that in no country in the world, women can fully benefit from the rights and opportunities men enjoy [28]. Therefore, gender inequality is also encountered in business life.

Due to the effect of social and cultural norms, lower level of education in women, and the cumulative effect of psychopathology, it is thought that occupational functionality levels are more affected by the female gender in the study data. In addition, it should be kept in mind that another important factor affecting occupational functionality is the economic and social situation (e.g., pandemic conditions) of the country where the study was conducted.

According to the results of the study, a significant difference was found between the groups with and without occupational functionality in terms of education level, and the rate of being a primary school graduate was found to be significantly higher in the group with occupational functionality. While evaluating these results, it may be necessary to evaluate the functionality in more detail in individuals who are primary school graduates, depending on the nature of the job and the functionality expected from the person. Occupational functionality expectations may be lower for individuals with lower education levels. In addition, it is known that cognitive processes are affected in OCD patients, and problems are detected in attention, memory, and visual-spatial functions, mainly in executive functions in terms of neuropsychology [29]. For this reason, it is thought that occupational functionality may be more affected by OCD patients who work in jobs that require higher education, knowledge, and skills.

When the data obtained in our study were examined, no difference was found between the two groups in terms of marital status and income level. The fact that the participants in both groups are currently employed may explain why no difference is observed in the average income level of the groups. However, many studies have reported low marriage rates in OCD patients [30]. For example, in the study of Mancebo et al., the rate of married people in the group with no occupational functionality was 27.8% [9]. In the study of Coban et al., the rate of married people in the group with no occupational functionality was 58.2%. In the current study, the rate of married individuals in the group with no occupational functionality was found to be 52%, which is consistent with the results of the study of Coban et al. [6]. The compatibility of the results of both studies can be associated with the inclusion of participants with similar average ages and the fact that the hospitals where the research was conducted are located in urban centers in Turkey.

In a study in the literature, OCD severity was found to be a dominant factor associated with occupational disability, followed by the severity of depressive symptoms and substance use disorder [9]. In another study, a comparison was conducted between clinical severity and professional competence, and they stated that lower depressive symptoms were associated with less occupational disability [6]. Similar to both studies, a significant difference was found in the current study in terms of total Y-BOCS scores.

It is an expected finding that occupational functionality levels were affected as a result of the increase in the severity of the disease. Parallel to previous studies, our study found no difference between the two groups in obsession scores. However, in the comparison of compulsion scores, it was found that the compulsion scores were significantly higher in the non-occupational functional group. Coban et al. identified this result by the fact that compulsions, characterized by actions rather than thoughts, cause more severe impairment during professional activity [6]. One of the factors that can be a measure of functionality may be the time spent with compulsions. From this point of view, low compulsion severity may be related to the deterioration of occupational functionality. As a result, the time spent by individuals during the struggle with the symptoms of the disease may be prolonged with the increase in the severity of the compulsions in which mental or motor responses are seen rather than obsessions in the form of impulses, thoughts, and images. It can be argued that it may impair functionality in all areas, especially by reducing occupational functionality.

The results of the present study should be evaluated within some limitations. First, in the cross-sectional study, information was not obtained about the total duration of being unemployed, and the qualitative and quantitative characteristics of the work needed to be examined in detail. The second limitation is that the evaluation of functionality is based on a verbal statement. Finally, the type of occupational activity, the total duration of the disorder, the medical history of the patients, and the effect of the previous psychotherapeutic interventions applied should have been discussed in the study.

## Conclusions

In previous literature, various studies on the effects of metacognition on psychopathology and functionality have been published. The present study is important because it is the first trial to elaborate on the effects of positive beliefs on occupational functionality in OCD patients, as it is one of the limited number of studies on occupational functionality. However, further studies are needed on the underlying causes of this effect. The results of the study contain important outcomes about the effects of metacognitions on functionality. These findings suggest that psychotherapeutic interventions, such as metacognitive therapy rooted in metacognition, may prove effective across various domains, encompassing clinical symptoms and occupational functioning, in the treatment of OCD.
